# Supplementary material for: Hermitian and non-Hermitian topology from photon-mediated interactions
Source: Nat Commun. 2024 Mar 16;15:2400. doi: 10.1038/s41467-024-46471-w (PMC10944496; doi:10.1038/s41467-024-46471-w)
Supplement: Supplementary file 1 — Supplementary Information [file 41467_2024_46471_MOESM1_ESM.pdf]

Supplementary Information:

Hermitian and Non-Hermitian Topology from Photon-Mediated Interactions

Federico Roccati*, Miguel Bello, Zongping Gong, Masahito Ueda, Francesco Ciccarello, Aurélia Chenu, Angelo Carollo

*Corresponding author. Email: federico.roccati@uni.lu

This Supplementary Information includes examples of the violation of the topological preservation and reversal in the fewer-emitters-than-resonators case, and the study of robustness against disorder for quantum emitters coupled to a QWZ photonic lattice.

This file includes:

- Supplementary text
- Figures S1, S2, S3
- Table S1
- Supplementary References

SUPPLEMENTARY TEXT

S1. Fewer-emitters-than-resonators case: violation of the topological correspondence

We consider here the most general case of a translationally invariant system of quantum emitters coupled to a photonic lattice. The interaction Hamiltonian within a unit cell can be written as

$$\hat{H}_{\text{int},n} = g\hat{\mathcal{S}}_n^\dagger \Pi \hat{\mathcal{A}}_n + \text{ H.c.}$$

Equation S1

where $\hat{H}_{\text{int}} = \sum_n \hat{H}_{\text{int},n}$, $\hat{\mathcal{A}}_n^{\text{T}} = \left(\hat{a}_{n1}, \dots, \hat{a}_{nN_b}\right)$ and $\hat{\mathcal{S}}_n^{\text{T}} = \left(\hat{\sigma}_{n1}, \dots, \hat{\sigma}_{nN_b}\right)$. The rank of the projector $\Pi = \text{diag}\left(p_1, \dots, p_{N_b}\right), p_i \in \{0,1\}$, specifies the number of quantum emitters per unit cell. In the main text, we had $\Pi = \mathbb{1}_{N_b}$.

The Bloch atomic Hamiltonian in this general case is as follows:

$$H_a(\mathbf{k}) = \Pi\left(\omega_e + \frac{g^2}{\omega_e - H_p(\mathbf{k})}\right)\Pi,$$

Equation S2

where only the nonzero block selected by Π is relevant. Importantly, when the lattice constant of the emitters' "superlattice" is larger than that of the photonic bath, the expression for the effective Hamiltonian can be used in this case, considering a suitably enlarged bath unit cell.

By considering specific setups, we concluded that, unlike the case of one emitter per resonator considered in the main text, no general statements can be made here. In particular, we show that a 1D Hermitian topological photonic lattice can induce both non-topological and topological interactions, according to the arrangement of the emitters (Fig. S1). Finally, we show how a non-Hermitian non-topological photonic lattice can mediate non-Hermitian topology at the atomic level (Fig. S2). These counterexamples support our claim.

Violation of Hermitian Topological Preservation

Consider a 1D photonic lattice whose Bloch Hamiltonian is

$$H_p(k) = [v + w\cos\, k]\tau_x + w\sin\, k\left[\cos\,(2\theta)\tau_y + \sin\,(2\theta)\tau_z\right],$$

Equation S3

with $v, w > 0, \theta \in [0, \pi/4]$. This two-band model corresponds to an SSH lattice for $\theta = 0$ and to a specific configuration of a Creutz ladder for $\theta = \pi/4$ (*17*). More precisely, $H_p(k) = U_\theta^\dagger H_{\text{SSH}}(k) U_\theta$, with $U_\theta = \cos\,\theta \mathbb{1}_2 + i\sin\,\theta\tau_x$ being a unitary transformation of the unit-cell modes and where $H_{\text{SSH}}(k)$ is the SSH Hamiltonian, Eq. (4) in the main text, in Fourier space. It belongs to the BDI symmetry class for any $\theta, U_{\text{TRS}} = \cos\,(2\theta)\mathbb{1}_2 - i\sin\,(2\theta)\tau_x, U_{\text{PHS}} = \tau_z$ and $U_{\text{CH}} = U_{\text{PHS}} U_{\text{TRS}}^*$ being the unitaries implementing antiunitary TRS and PHS, and unitary chiral symmetry, respectively (*1*,*2*).

The original two-site photonic unit cell $(\hat{a}_{n,1}, \hat{a}_{n,2})$ can be formally enlarged to a four-site cell by relabeling as follows: $(\hat{a}_{n,1}, \hat{a}_{n,2}, \hat{a}_{n+1,1}, \hat{a}_{n+1,2}) \to (\hat{a}_{n,1}, \hat{a}_{n,2}, \hat{a}_{n,3}, \hat{a}_{n,4})$. A two-band atomic Hamiltonian is then obtained by coupling only two quantum emitters to two resonators in this enlarged photonic unit cell. There are only two nonequivalent ways to do so. We set $\omega_e = \omega_0 = 0$ to preserve chiral symmetry (see main text).

(i) The on-cell case corresponds to coupling the quantum emitters to the resonators in sublattices 1 and 2, *i.e.*, $\Pi = \text{diag}\,(1,1,0,0)$. Since $[\Pi, U_s] = 0$, for $s \in \{\text{TRS}, \text{PHS}, \text{CH}\}$, the atomic subsystem inherits all the symmetries of the photonic one. Besides belonging to the same symmetry class, the atomic and photonic systems have the same topology, as observed in the one-emitter-per-resonator case. Indeed, by highlighting the dependence on the parameters in the Bloch photonic Hamiltonian $H_{\text{SSH}}(k; v, w)$, one can see that $H_a(k) \propto [H_{\text{SSH}}(k; v^2, -w^2)]^{-1}$. Thus, the atomic and photonic systems possess the same winding number (*3*). Note that it is sufficient to analyze the $\hat{H}_a$ obtained in the SSH case ($\theta = 0$) since the other cases ($\theta \neq 0$) are unitarily equivalent.

(ii) The cell-breaking case corresponds to coupling quantum emitters to the resonators in sublattices 2 and 3, *i.e.*, $\Pi = \mathrm{diag}\,(0,1,1,0)$. The atomic Hamiltonian inherits PHS, with $U_{\mathrm{PHS}} = -\tau_z$ for any θ, but breaks TRS and chiral symmetry, except for $\theta = 0$. For an SSH photonic lattice ($\theta = 0$), the mediated Hamiltonian is topological when the photonic one is not, and vice versa. Indeed, $H_a(k) \propto [H_{\mathrm{SSH}}(k; w^2, -v^2)]^{-1}$; therefore, the photonic winding number is zero (nonzero) when the atomic one is nonzero (zero). This is reminiscent of the change in topology following a redefinition of the intra/intercell coupling amplitudes. For $0 < \theta < \pi/4$, the mediated Hamiltonian belongs to the D symmetry class, with only PHS, while for $\theta = \pi/4$ the mediated atomic Hamiltonian is gapless for any v and w. The gap is $\Delta = 2g^2 w \cos\,(2\theta)/(v^2 + w^2)$. It is thus meaningless to compare the photonic and atomic topologies in these cases.

Violation of Non-Hermitian Topological Reversal

Similar to the Hermitian case, if the number of emitters is less than the number of resonators, no general topological reversal or preservation can be claimed. Stacking two topological photonic lattices with opposite topological invariants and coupling only one of them to emitters in the one-emitter-per-resonator manner is a general method for constructing counterexamples. The emitters' Hamiltonian will be nontrivial owing to either topological reversal or preservation with respect to the sublattice with which they interact. If the coupling between the two photonic lattices is not so intense such that the emitters' Hamiltonian contains a gap, the nontrivial emitters' topology persists even if the coupling between the two lattices is activated. This holds for Hermitian systems as well.

As a simple (counter) example, we constructed a 1D non-Hermitian photonic bath with zero spectral winding number that nevertheless induces a nonzero spectral winding number for the emitters' Hamiltonian. The bath consists of two unidirectional Hatano–Nelson chains with opposite chiralities and Hermitian interchain hopping. The emitters are only coupled to one of them, say the leftward one, see Fig. S2. Then, we can write the photonic Bloch Hamiltonian and projector as follows:

$$H_p(k) = \begin{bmatrix} \gamma\left(e^{ik} - i\right) & J \\ J & \gamma\left(e^{-ik} - i\right) \end{bmatrix}, \qquad \Pi = \begin{bmatrix} 1 & 0 \\ 0 & 0 \end{bmatrix}.$$

Equation S4

We assume $J < \gamma$ so that $H_p(k)$ is gapped with respect to $\omega_e = -i\gamma$. Substituting the above expressions (S4) into Eq. (S2), we obtain

$$H_a(k) = -\frac{g^2\gamma}{\gamma^2 - J^2} e^{-ik} - i\gamma,$$

Equation S5

which is characterized by a nonzero spectral winding number $\nu_a = -1$ with respect to the base energy ω_e (*4*). To visualize such a breakdown of the non-Hermitian topological

reversal in this model, we consider a finite system under the open boundary conditions and remove some emitters near the boundaries. As shown in Fig. S2, we find that the skin effect occurs in the emitter's array, though the photonic bath does not exhibit a skin effect.

S2. Robustness against disorder

A standard way to study the impact of disorder is the so-called *diagonal disorder* (*5*), *i.e.*, a diagonal perturbation to the Hamiltonian in Eq. (S6), whose entries are random numbers with zero mean. Another option, perhaps more relevant for the systems under investigation, is what we could call *frequency disorder, i.e.*, to use the expression for the atomic Hamiltonian

$$\hat{H}_a = (\mathbb{1}_{N_c} \otimes \Pi)\left(\omega_e + \frac{g^2}{\omega_e - \hat{H}_p}\right)(\mathbb{1}_{N_c} \otimes \Pi)\,,$$

Equation S6

that is the real space version of Eq. (S2), replacing ω_e with $\omega_e + \delta\omega_i$ with $\delta\omega_i$ randomly distributed around zero, for $i = 1, \dots, N$ (note that ω_e does not appear only in the diagonal of $\hat{H}_a$). To study the robustness of the topological phase, we can use the Kitaev sum (*6*), and compare diagonal and frequency disorder. As an example, in Fig. S3 (below), we show that both kinds of disorders have a similar effect for the QWZ case [Eq. (5) in the main text], which seems to suggest that the changes in the effective couplings induced by the changes in the transition frequency have a negligible effect. Qualitatively, a perturbation in the frequency $\delta\omega$ leads to a perturbation in the effective couplings $\sim g^2\delta\omega/J$.

FIGURES S1, S2, S3

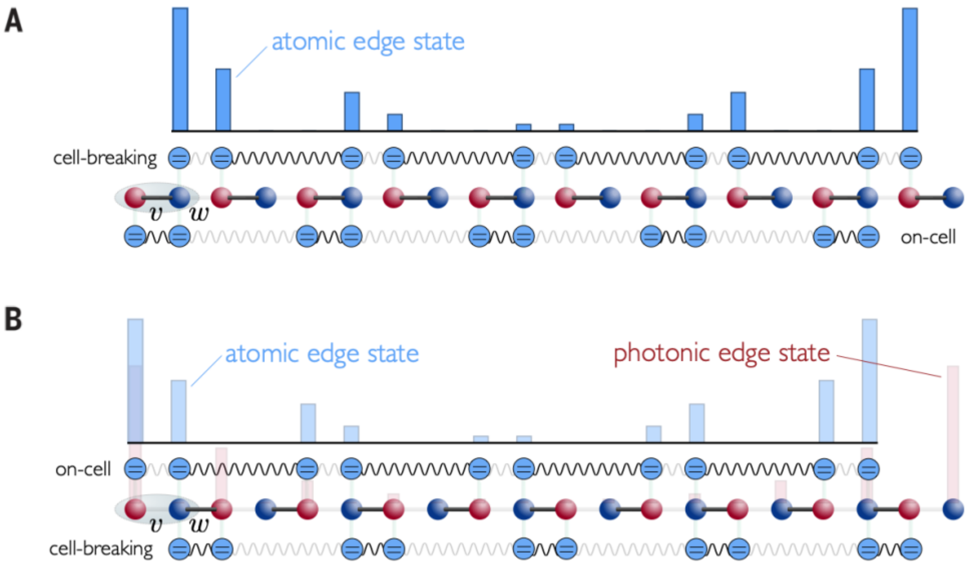

Fig. S1. Violation of the Hermitian topological preservation. (**A**) Photonic lattice as in Eq. (S3) in real space under open boundary conditions in the nontopological phase $v > w$. Quantum emitters are coupled to it in the on-cell and cell-breaking configurations below and above the photonic lattice, respectively. In the former case, the atomic system inherits all symmetries and topology of the photonic lattice. In the latter case, a pair of topologically protected atomic edge states appear (only one is shown in cyan) even without photonic topology. For $\theta = 0$, the atomic system has chiral symmetry and belongs to the BDI symmetry class, and its topological invariant is the winding number. Wiggly lines sketch the mediated couplings between quantum emitters, and their thickness represents the strength. In the almost-fully dimerized limit ($v \gg w$), the atomic system follows the photonic dimerization in the on-cell configuration, while it does not in the cell-breaking one. This leads to the emergence of the edge states. (**B**) Same as **A**, with a topological photonic lattice, with on-cell and cell-breaking configurations present above and below the photonic lattice, respectively. Here, the photonic edge states (shaded red) are accompanied by atomic ones (shaded cyan) in the on-cell configuration. Parameter values for the edge states are as follows: $\omega_e = 0, \theta = 0$ (a different θ does not substantially affect their profile), $w/v = 4/5$ in **A**, $v/w = 2/5[4/5]$ in **B** for the photonic [atomic] one.

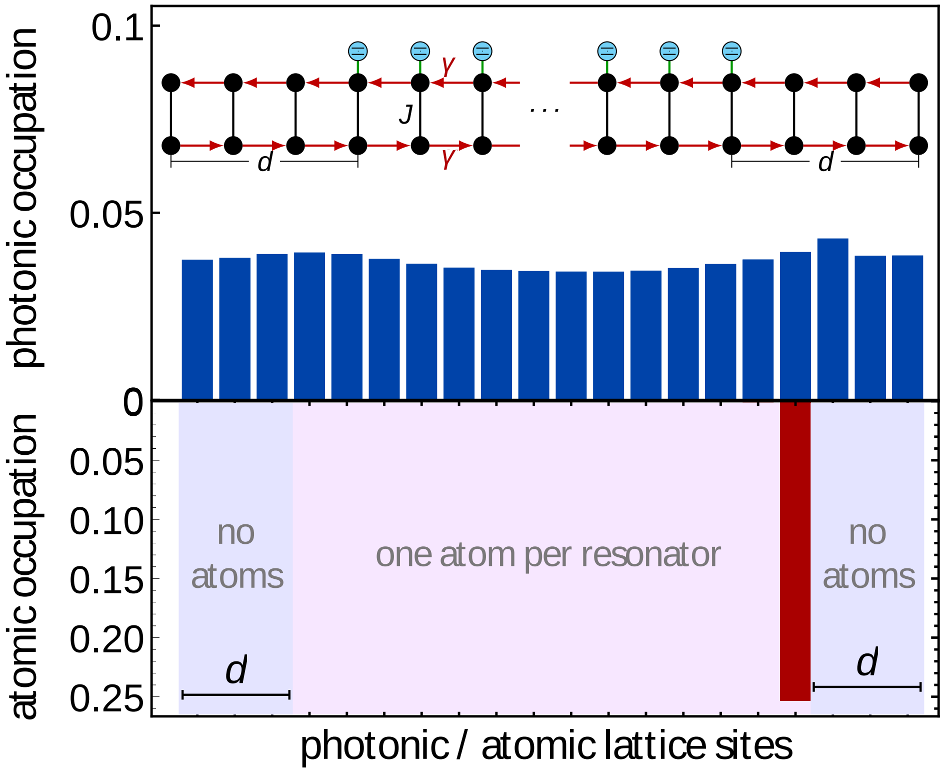

Fig. S2. Violation of the non-Hermitian topological reversal. The photonic bath is built from two coupled unidirectional Hatano–Nelson chains of $2N$ sites with opposite chiralities. Under open boundary conditions, only the resonators in the upper chain are coupled to the emitters, except for the leftmost and rightmost d sites. Note that a background loss captured by an imaginary shift $-i\gamma$ is omitted in the inset. The atomic skin effect (bottom) occurs despite the lack of a photonic skin effect (top). Both figures show $\overline{|\langle n \mid \psi_m \rangle|^2}$ with n being an atomic or photonic site and $\overline{\cdots}$ being the average over all the normalized right eigenstates $|\psi_m\rangle$. On the photonic side, we further sum up the pairs of sites in the same unit cell, *i.e.*, those with the same horizontal positions. Parameters: $N = 20, d = 3, J = 0.5\gamma$, and $g = 0.1\gamma$.

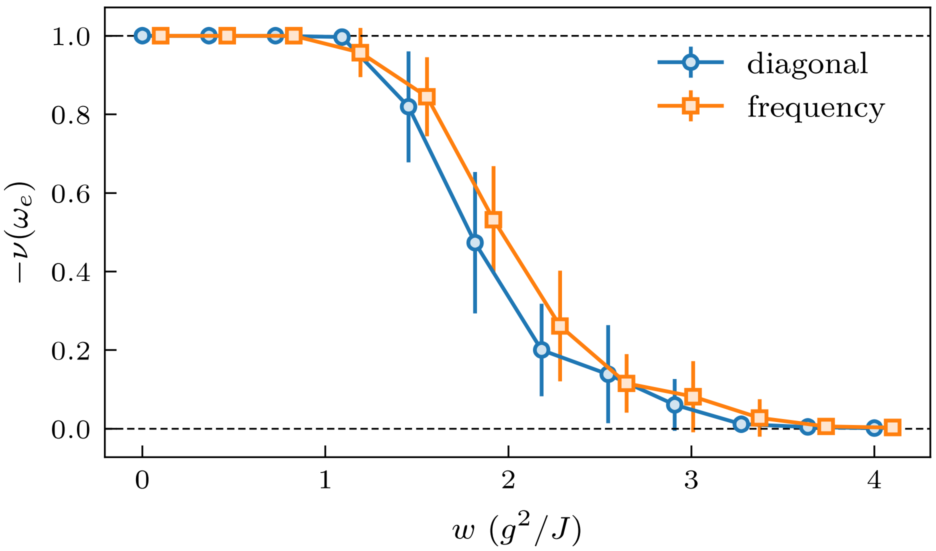

Fig. S3. Comparing diagonal and frequency disorder. Kitaev sum for a set of emitters coupled to the QWZ photonic lattice [Eq. (5) in the main text], as a function of the disorder strength, for two different types of disorder. The elements of the diagonal perturbation, in one case, and the frequency shifts, in the other, have been chosen uniformly distributed over the interval $[-w, w]$. The computation has been performed for a lattice with 30×30 unit cells, periodic boundary conditions, and the same number of emitters and resonators. The dots correspond to the average value over 12 different disorder realizations, while the error bars indicate one standard deviation. The points in the case of frequency disorder are slightly displaced to the right for an easier comparison. We note that in the y-axis, "ν" represents the Kitaev sum (not any of the topological invariants mentioned in the main text).

TABLE S1

Class	Symmetry T	C	S	Spatial dimension 1	2	3
A	0	0	0	0	$\mathbb{Z}$	0
AIII	0	0	1	$\mathbb{Z}$	0	$\mathbb{Z}$
AI	+	0	0	0	0	0
BDI	+	+	1	$\mathbb{Z}$	0	0
D	0	+	0	$\mathbb{Z}_2$	$\mathbb{Z}$	0

Table S1. Periodic table of topological insulators. The Altland–Zirnbauer classification of Hermitian photonic topological insulators based on the presence (absence) of time reversal (T), particle–hole (C), and chiral (S) symmetries, denoted by 1 or $+(0)$. The + sign indicates that the square of T (or C) is 1, $T^2 = 1$ (*7*). The black arrows highlight the non-resonant transitions between classes of photonic and atomic symmetry, cf. main text. Under the spatial dimension, the entries $\mathbb{Z}, \mathbb{Z}_2, 0$ represent the possible value of the relevant topological invariant. The table was adapted with permission from (*2*). Copyrighted by the American Physical Society.

Supplementary References

[1] T. Ozawa, H. M. Price, A. Amo, N. Goldman, M. Hafezi, L. Lu, M. C. Rechtsman, D. Schuster, J. Simon, O. Zilberberg, I. Carusotto, Topological photonics. *Rev. Mod. Phys.* **91**, 015006 (2019).

[2] C. K. Chiu, J. C. Y. Teo, A. P. Schnyder, S. Ryu, Classification of topological quantum matter with symmetries. *Rev. Mod. Phys.* **88**, 035005 (2016).

[3] J. K. Asbóth, L. Oroszlány, A. Pályi. *Lect. Notes Phys.* **919**, 166 (2016).

[4] Z. Gong, Y. Ashida, K. Kawabata, K. Takasan, S. Higashikawa, M. Ueda, Topological Phases of Non-Hermitian Systems. *Phys. Rev. X* **8**, 031079 (2018).

[5] E. Prodan, T. L. Hughes, and B. A. Bernevig, Entanglement spectrum of a disordered topological chern insulator. *Phys. Rev. Lett.* **105**, 115501 (2010).

[6] A. Kitaev, Anyons in an exactly solved model and beyond. *Annals of Physics* **321**, January Special Issue, 2–111 (2006).

[7] A. Altland, M. R. Zirnbauer, Nonstandard symmetry classes in mesoscopic normal-superconducting hybrid structures. Phys. Rev. B **55**, 1142 (1997).
